# Development of Triiodothyronine Polymeric Nanoparticles for Targeted Delivery in the Cardioprotection against Ischemic Insult

**DOI:** 10.3390/biomedicines9111713

**Published:** 2021-11-18

**Authors:** Ozlem Ozen Karakus, Noureldien H. E. Darwish, Thangirala Sudha, Taher A. Salaheldin, Kazutoshi Fujioka, Peter C. Taylor Dickinson, Brian Weil, Shaker A. Mousa

**Affiliations:** 1 The Pharmaceutical Research Institute, Albany College of Pharmacy and Health Sciences, Rensselaer, NY 12144, USA; Ozlem.Karakus@acphs.edu (O.O.K.); noureldien.darwish@acphs.edu (N.H.E.D.); sudha.thangirala@acphs.edu (T.S.); taher.salaheldin@acphs.edu (T.A.S.); Kazutoshi.Fujioka@acphs.edu (K.F.); 2 Clinical Pathology (Hematology Section), Faculty of Medicine, Mansoura University, Mansoura 35516, Egypt; 3 Pro-Al Medico Technologies Inc., Suffern, NY 10901, USA; dickinsonpct@gmail.com; 4 Departments of Physiology & Biophysics, School of Medicine & Biomedical Sciences, University at Buffalo, Buffalo, NY 14203, USA; bweil@buffalo.edu; 5 VA WNY Healthcare System, Buffalo, NY 14215, USA

**Keywords:** thyroid hormone, triiodothyronine, Nano-T3, cardiac arrest, ischemia, phosphocreatine, resuscitation

## Abstract

Ischemic heart disease is the main cause of death globally. Cardioprotection is the process whereby mechanisms that reduce myocardial damage, and activate protective factors, contribute to the preservation of the heart. Targeting these processes could be a new strategy in the treatment of post-ischemic heart failure (HF). Triiodothyronine (T3) and thyroxine (T4), which have multiple effects on the heart, prevent myocardial damage. This study describes the formulation, and characterization, of chemically modified polymeric nanoparticles incorporating T3, to target the thyroid hormone receptors. Modified T3 was conjugated to polylactide-co-glycolide (PLGA) to facilitate T3 delivery and restrict its nuclear translocation. Modified T3 and PLGA-T3 was characterized with ^1^H-NMR. The protective role of synthesized phosphocreatine (PCr) encapsulated PLGA-T3 nanoparticles (PLGA-T3/PCr NPs) and PLGA-T3 nanoparticles (PLGA-T3 NPs) in hypoxia-mediated cardiac cell insults was investigated. The results showed that PLGA-T3/PCr NPs represent a potentially new therapeutic agent for the control of tissue damage in cardiac ischemia and resuscitation.

## 1. Introduction

Cardioprotection is a wide-ranging term that includes all mechanisms for the protection of the heart, and the multiple factors involved, such as cardiomyocytes, cytokines, cell growth, angiogenesis, and mitochondria. Many studies have shown that thyroid hormone plays an important role in cardioprotection, and its regulating mechanisms [1,2]. The major thyroid hormone produced by the thyroid gland is L-thyroxine (T4), and when one of the iodines is removed by iodothyronine deiodinase enzymes, it forms L-triiodothyronine (T3).

T3, the most active form of thyroid hormone, is involved in cardiac protection through several mechanisms. One of these classical mechanisms is by direct genomic signaling. When T3 binds to its nuclear receptors, by coupling to T3 response elements (TRE) in the promoter regions of target genes, it regulates transcription [3]. In addition, through non-genomic actions it interacts with cytoplasmic and membrane-associated thyroid hormone receptors, namely the integrin, αvβ3, and mediates the action of thyroid hormone on the ion channels located in the cardiomyocyte membrane [4,5], to protect cells from ischemic injury [6]. By this action, T3 activates the cell surface receptor integrin αvβ3 and also leads to the formation of new blood vessels (angiogenesis).

Fang et al. showed that T3 treatment improves cardioprotection against ischemia reperfusion injury in isolated rat heart cells by preserving major Ca^2+^ cycling proteins [7]. Forini et al. showed that T3 treatment of isolated rat heart cells can prevent further mitochondrial disturbance and damage in ischemia [8]. They also described how T3 protects against the oxidative stress condition, by limiting p53 upregulation [9].

Thus, T3 emerges as an attractive novel therapeutic strategy in post-ischemic heart failure (HF) [10,11]. Although T3 appears to be a promising treatment, it has a short half-life in blood, and its genomic actions can have potentially deleterious effects. Nano-targeted delivery of T3 may prolong the circulation half-life, restrict its nuclear translocation, enhance delivery to a desired site (αvβ3), and improve its safety profile [12]. Additionally, T3 conjugated polymeric nanoparticles (NPs) allow for the encapsulation of different bioactive molecules such as phosphocreatine (PCr), which is a high-energy phosphate for the myocardium, and other organs. Among polymeric NPs, polylactic acid-co-glycolic acid NPs (PLGA NPs) may play an important role in delivering hydrophobic therapeutics to the targets, such as tumor tissues. PLGAs are hydrophobic, biodegradable, FDA-approved polymers that are physically strong and highly biocompatible with free drugs, as well as other molecules such as peptides [13,14].

A critical mechanism through which hypoxia/ischemia causes irreversible myocardial injury is by the exhaustion of adenosine triphosphate (ATP). Creatine (Cr), cyclocreatine (CCr) and their water-soluble salts creatine-phosphate/Phosphocreatine (CrP/PCr), and cyclocreatine-phosphate (CCrP) are potent bioenergetic agents that preserve high levels of ATP during ischemia. These can be encapsulated into T3 conjugated NPs. PCr is an important component in the intracellular system of energy buffering, and transfer, and provides for the high energy demands of the heart [15]. Under normal conditions, Cr is synthesized in the brain and used to form PCr through creatine kinase. PCr donates its phosphate group to adenosine diphosphate (ADP) to resynthesize adenosine triphosphate (ATP) under conditions of ATP exhaustion [16,17]. Thus, PCr allows ATP synthesis even in the absence of oxygen and glucose [18].

Supplementation with PCr may be potentially beneficial to patients with acute and chronic myocardial ischemic injury [15,19]. This study presents the synthesis and investigation of novel PLGA-T3 NPs with, or without, the encapsulation of PCr for potential benefits against cardiac ischemic insults.

## 2. Experimental Section

### 2.1. Chemicals and Analysis

4-Hydroxybenzylamine, di-*tert*-butyl di-carbonate, HCl (4 N in dioxane), N,N′-Di-Boc-1H-pyrazole-1-carboxamidine, TEA, sodium ascorbate, and copper sulfate were purchased from Sigma-Aldrich (St. Louis, MO, USA). PLGA5k-NHS was purchased from Nano Soft Polymers (Winston-Salem, NC, USA). All commercially available chemicals were used without further purification. Chemical structures of all synthesized compounds were confirmed with ^1^H-NMR, ^13^C NMR, and mass spectrometry. The NMR experiments were all performed on a Bruker Advance II 600 MHz spectrometer at the Center for Biotechnology and Interdisciplinary Studies, Rensselaer Polytechnic Institute (Troy, NY, USA). Thin layer chromatography was performed on silica gel plates with fluorescent indicators. Mass spectral analysis was performed on either an Applied Biosystems API4000 LC/MS/MS or Advion Expression CMC^L^ mass spectrometer. Analytical HPLC analyses were performed with a Waters 2695 system equipped with a Pursuit XRs C18 column (150 × 4.6 mm), with a flow rate of 1 mL/min, using a gradient of MeOH and water (0.1% TFA), and UV detection at 227 and 254 nm.

### 2.2. 2-((benzyloxy)carbonyl)amino)-3-(4-(4-hydroxy-3-iodophenoxy)-3,5 di-iodophenyl) Propanoic Acid ***(2)***

T3 (compound **1**) (1 g, 1.53 mmol, 1 eq) and NaHCO_3_ (0.39 g, 4.59 mmol, 3 eq) were mixed in 20 mL THF: water (1:1) and benzyl chloroformate (0.31 g, 1.84 mmol, 1.2 eq) was added to a suspension at room temperature. After stirring for 4 h, solvents were removed under reduced pressure, and the residue was purified by column chromatography. Yield: 1.20 g, 87%.

^1^**H-NMR (600 MHz, DMSOd6):** 2.82 (1 H, dd, CH_2_), 3.11 (1 H, dd, CH_2_), 4.25 (1 H, td, CH), 5.03 (2H, s, -OCH_2_), 6.60 (1H, dd, ArCH), 6.84 (1H, d, ArCH), 7.03 (1H, d, ArCH), 7.33 (3H, t, ArH-Cbz), 7.38 (2H, t, ArH-Cbz), 7.74 (1H, d, NH), 7.88 (2H, s, ArH), 10.04 (1H, s, Ar-OH), and 12.89 (1H, s, COOH). ^13^**C-NMR (150 MHz,** **DMSOd6):** 35.2, 55.4, 65.9, 84.9, 92.3, 115.6, 116.5, 125.1, 127.9, 128.2, 128.8, 137.4, 139.8, 141.1, 149.5, 156.3, and 173.2. **MS (ESI+):** C_23_H_18_I_3_NO_6_+H^+^ Calcd, 785.83 Anal. Calcd. (%): C, 35.19; H, 2.31; N, 1.78. Found (%): C, 35.11; H, 2.22; and N, 1.39.

### 2.3. Methyl2-(benzyloxy)carbonyl)amino)-3-(4-(4-hydroxy-3-iodophenoxy)-3,5-diiodophenyl) Propanoate ***(3)***

SOCl_2_ (5 mL) was slowly added to a solution of compound **2** (1 g, 1.27 mmol) in MeOH (15 mL) at 0 °C. The reaction mixture was refluxed at 70 °C for 2 h, before cooling it to room temperature. MeOH was removed in vacuo and the resulting residue was poured onto ice-H_2_O (25 mL) and extracted with DCM (2 × 10 mL). The combined organic extracts were washed with 10% NaHCO_3_ (2 × 10 mL), and brine, and dried (Na_2_SO_4_), and concentrated to provide the product as a white solid. Yield: 1.02 g, 85%. 

^1^**H-NMR (600 MHz, DMFd6):** 3.03 (1 H, dd, CH_2_), 3.24 (1 H, dd, CH_2_), 3.52 (3H, s, -OCH_3_), 4.53 (1 H, td, CH), 5.09 (2H, s, -OCH_2_), 6.69 (1H, dd, ArCH), 6.99 (1H, d, ArCH), 7.15 (1H, d, ArCH), 7.36 (3H, t, ArH-Cbz), 7.41 (2H, t, ArH-Cbz), 7.82 (1H, d, NH), 8.01 (2H, s, ArH), and 10.39 (1H, s, Ar-OH). ^13^**C-NMR (150 MHz, DMFd6):** 35.3, 51.8, 55.5, 65.9, 83.9, 91.2, 115.2, 116.4, 125.2, 127.6, 127.8, 128.5, 137.3, 139.3, 141.3, 149.6, 152.7, 156.4, 172.0. **MS (ESI+):** C_24_H_20_I_3_NO_6_+H^+^ Calcd, 799.84 Anal. Calcd. (%): C, 36.07; H, 2.52; N, 1.75. Found (%): C, 36.23; H, 2.39; and N, 1.66.

### 2.4. Methyl2-(benzyloxy)carbonyl)amino)-3-(4-(4-(3-(tert-butoxycarbonyl)amino) propoxy)-3-iodophenoxy)-3,5-diiodophenyl) Propanoate ***(4)***

K_2_CO_3_ (275 mg, 1.99 mmol, 3 eq) was added with stirring to a solution of compound **3** (530 mg, 0.66 mmol, 1 eq) in ACN (25 mL) at room temperature. After the reaction mixture was stirred for 30 min, tert-butyl (3-bromopropyl) carbamate (0.19 g, 0.79 mmol, 1.2 eq) was added to the mixture and the temperature was increased to 80 °C and refluxed for 24 h. The solution was filtered to remove excess K_2_CO_3_. Solvents were removed under reduced pressure, and the oily residue was purified by column chromatography. Yield: 536 mg, 85%.

^1^**H-NMR (600 MHz, CDCl_3_):** 1.46 (9H, s, -CH_3_), 2.05 (2H, m, -CH_2_-), 3.01 (1 H, dd, -CH_2_), 3.11 (1 H, dd, -CH_2_), 3.42 (2H, t, -CH_2_-), 3.79 (3H, s, -OCH_3_), 4.03 (2H, t, -CH_2_-), 4.66 (1 H, td, -CH), 5.15 (2H, s, -OCH_2_), 5.42 (1H, s, NH), 6.68 (1H, dd, ArCH), 6.73 (1H, d, ArCH), 7.28 (1H, d, ArH), 7.35 (1H, d, ArH-Cbz), 7.38 (2H, d, ArH-Cbz), 7.39 (2H, d, ArH-Cbz), and 7.66 (2H, s, ArH). ^13^**C-NMR (150 MHz, CDCl_3_):** 28.4, 29.2, 36.9, 38.5, 52.6, 54.7, 67.2, 68.2, 79.0, 86.7, 90.9, 112.0, 115.7, 126.5, 128.1, 128.3, 128.6, 136.0, 137.1, 141.0, 150.5, 152.9, 155.5, 156.0, and 171.3. **MS (ESI+):** C_32_H_35_I_3_N_2_O_8_+H^+^ Calcd, 956.95 Anal. Calcd. (%): C, 40.19; H, 3.69; N, 2.93. Found (%): C, 40.25; H, 3.54; and N, 2.79.

### 2.5. 3-(4-(4-(3-aminopropoxy)-3-iodophenoxy)-3,5-diiodophenyl)-2 ((benzyloxy)carbonyl)amino) Propanoic Acid ***(5)***

LiOH (173 mg, 7.2 mmol) was added to a mixture of compound **4** (400 mg, 0.42 mmol) in 20 mL THF: water (1:1) and stirred 2 h at room temperature. The mixture was acidified to pH = 5 with HCl (1 M), extracted with DCM (2 × 100 mL), and washed with water (100 mL) and then brine. The solution was dried over magnesium sulfate, filtered, and concentrated to give a white solid. Yield: 468 mg, 85%. Crude product (325 mg, 0.57 mmol) was dissolved in 3 mL anhydrous 1,4-dioxane and 3 mL HCl (4 N in dioxane) and stirred at room temperature. After 24 h, the solvent was removed under reduced pressure, and the oily residue was precipitated by stirring in diethyl ether to afford compound **5** as a yellowish solid. Yield 240 mg, 90%.

^1^**H-NMR (600 MHz, DMSOd6):** 2.10 (2H, m, -CH_2_-), 2.81 (1 H, dd, -CH_2_-), 2.88 (1 H, m, -CH_2_), 3.00 (2H, t, -CH_2_-), 4.09 (2H, t, -CH_2_-), 4.25 (1 H, td, -CH), 5.01 (2H, s, -OCH_2_), 6.68 (1H, dd, ArCH), 6.98 (1H, d, ArCH), 7.14 (1H, d, ArH), 7.31 (3H, m, ArH-Cbz), 7.37 (2H, m, ArH-Cbz), 7.70 (1H, s, NH), and 7.89 (2H, s, ArH). ^13^**C-NMR (150 MHz,** **DMSOd6):** 27.2, 30.3, 35.2, 36.5, 42.8, 55.6, 65.8, 66.6, 87.6, 92.5, 113.6, 116.1, 125.5, 127.8, 128.2, 128.8, 137.4, 140.1, 141.0, 150.8, 152.0, 152.7, 156.4, and 173.1. **MS (ESI+):** C_26_H_25_I_3_N_2_O_8_+H^+^ Calcd, 841.88 Anal. Calcd. (%): C, 37.08; H, 2.99; N, 3.33. Found (%): C, 37.15; H, 2.54; and N, 3.48. 

### 2.6. PLGA-T3-Cbz ***(6)*** and PLGA-T3 ***(7)***

Compound **5** (100 mg, 1 eq) and 1 eq of PLGA5k-NHS were dissolved in 5 mL DMSO (4:1) and stirred for 24 h at rt. to form Compound **6**. Compound **6** was then dialyzed against water using a membrane (MWCO 1kDa) for 2 days, Yield: 523 mg, 60%. 

2 mL HBr (in 33% AcOH) was added to compound **6** (100 mg, 1 eq) in 5 mL DCM and stirred at rt for 1 h. After removing the solvent, crude compound **7** was dialyzed against water using a membrane (MWCO 1kDa) for 2 days. Yield: 600 mg, 60%.

^1^**H-NMR (600 MHz, DMSOd6):** 2.10 (2H, m, -CH_2_-), 2.81 (1 H, dd, -CH_2_-), 2.88 (1 H, m, -CH_2_), 3.00 (2H, t, -CH_2_-), 4.09 (2H, t, -CH_2_-), 4.25 (1 H, td, -CH), 5.01 (2H, s, -OCH_2_), 6.68 (1H, dd, ArCH), 6.98 (1H, d, ArCH), 7.14 (1H, d, ArH), 7.31 (3H, m, ArH-Cbz), 7.37 (2H, m, ArH-Cbz), 7.70 (1H, s, NH), and 7.89 (2H, s, ArH).

### 2.7. Synthesis and Characterization of PLGA-T3 NPs

**Figure 1:**
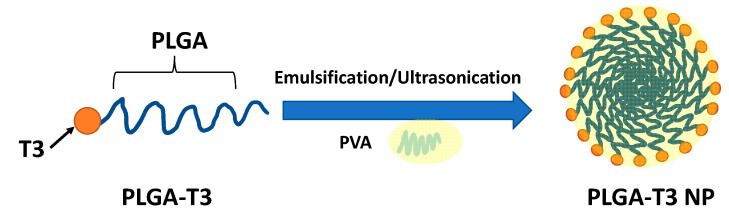
Schematic representation of PLGA-T3 NP preparation.

PLGA-T3 NPs were synthesized by a modified spontaneous emulsification solvent diffusion method (SESD) in which they were effectively obtained by forming a nanoscale shell made from nontoxic polyvinyl alcohol (PVA) around PLGA-T3 under ultrasonication and mechanical stirring (Figure 1) [20].

The typical operating procedure was as follows; 200 mg of PLGA-T3 was dissolved in 1 mL DMSO. The obtained polymer solution was then added (20 uL/min) into 40 mL of aqueous 1% PVA solution in a 100-mL glass flask under ultrasonication using prob sonicator at 100 W for 10 min while continuously stirring at 600 rpm for 2 hrs. The prepared nanoparticles were collected and purified by washing three times with purified water (distilled water filtered by 0.2 µm Millipore filter) to remove DMSO and unformulated precursors using centrifugation tubes at 8000 rpm for 2 h. After centrifugation, A pellet of PLGA-T3 NPs precipitated at the bottom of the centrifuge tube and the supernatant containing DMSO and unformulated precursors was discarded. The PLGA-T3 NPs pellet was re-dispersed in 5 mL purified water for further characterization. The aqueous dispersed PLGA-T3 NPs were then freeze-dried in a vacuum to obtain powdered NPs. A schematic representation of nanoparticle preparation is given in Figure S11 in the supplementary materials.

### 2.8. The Synthesis of PLGA-T3/PCr NPs

Phosphocreatine (PCr) encapsulated PLGA-T_3_ nanoparticles were synthesized by a modified combined double emulsion method in which PLGA-T3/PCr NP were effectively obtained by forming nanoscale shells made from PVA around PLGA-T3 NPs/PCr under ultrasonication and mechanical stirring [20,21]. The typical operating procedure was as follows; 200 mg of PLGA-T3 and 45 mg PCr were dissolved in 1 mL DMSO. The obtained polymer solution was then added (20 uL/min) into 20 mL of aqueous 1% PVA solution under continuous stirring, at 600 rpm, for 2 hrs. This solution was then added to 50 mL 0.5% PVA under stirring for 24 h. PLGA-T3/PCr NP were harvested, and washed three times, by centrifugation at 8000 rpm, and re-dispersed in 5 mL purified water for further characterization. The aqueous dispersed PLGA-T3/PCr NPs were then freeze-dried in a vacuum to obtain the powdered NPs.

### 2.9. The Characterization of the Prepared PLGA-T3/PCr NPs

A zeta sizer (Malvern Instrumentation Co., Westborough, MA, USA) was used to determine the size distribution and zeta potential of the nano capsules in the aqueous dispersion. A high resolution transmission electron microscope (HRTEM) was used for topographic imaging. A total of 20 µL of a diluted NP dispersion was placed on 300 mesh carbon coated grid, left to dry for 15 min, then fixed on the platinum sample holder for TEM imaging under 200 KV. The T3 concentration was measured by HPLC.

### 2.10. Encapsulation Efficiency (EE) and Loading Ratio (LR)

The encapsulation efficiency was calculated by analyzing the T3 loading in the NPs. After lyophilization, the weighed NP powder was dispersed in 3 mL of DMSO for 10 min. The amount of T3 in the DMSO was determined by using UV absorption of T3 at 295 nm with calibration curve (Figure S11).

$$\mathrm{Encapsulation}\;\mathrm{Efficacy}\;(\mathrm{EE})=\frac{T3\;concentration\;in\;NP}{Intial\;T3\;concentration}\;\times \;100$$

The loading ratio of the PLGA-T3 NPs was determined by measuring the amount of T3 in the nanoparticles, and the weight of the whole nanoparticle [21].

$$\mathrm{Loading}\;\mathrm{Efficiency}\;=\;\frac{T3\;concentration\;in\;NP}{Total\;weight\;of\;NP}\;\times \;100$$

### 2.11. In Vitro Release Study

The release kinetics studies were performed in FBS and PBS. A known amount of the PLGA-T3/Pcr NPs was suspended in 15 mL of PBS/FBS. The solutions were incubated at 37 °C. At pre-determined time intervals over a week, 500 µL of the solution was filtered through millipore tubes containing a 30 kD membrane to separate the released PCr from the NPs and was analyzed by liquid chromatography-electrospray ionization-tandem mass spectrometry (LC-MS/MS) [22].

### 2.12. Evaluation of the Cardioprotective Effect of T3 and PLGA-T3/PCr NPs under Hypoxia

The cardioprotective effect of T3 and PLGA-T3/PCr NPs under hypoxia were studied using isolated neonatal mice cardiomyocytes using a Pierce™ primary cardiomyocyte isolation kit (ThermoFisher, Waltham, MA, USA). The isolation of neonatal mice cardiomyocytes was performed as previously described [23], and all methods described in this protocol were approved by the Animal Care and Use Committee (IACUC), and adhered to federal, and state, regulations.

All steps were performed in a sterile laminar flow cell culture hood. This protocol is intended for the isolation of neonatal mouse hearts from approximately 8–10 pups. Briefly, freshly dissected neonatal hearts were immediately placed into separate 1.5 mL sterile microcentrifuge tubes with 500 µL ice cold Hanks’ balanced salt solution (HBSS). Each heart was minced into 1–3 mm^3^ pieces and washed twice with ice cold HBSS to remove blood from the tissue. The cardiomyocytes were incubated with cardiomyocyte isolation enzymes in a 37 °C incubator for 30 min.

After washing, complete Dulbecco’s modified eagle medium (DMEM) for primary cell isolation was added to each tube and the solutions were pipetted up and down using a sterile 1.0 mL pipette tip. After the tissue was primarily a single-cell suspension, an additional 1.0 mL of complete DMEM was added for primary cell isolation to each tube to bring the total volume to 1.5 mL. After the evaluation of the cell concentration and viability, the cells were allowed to grow in 35 mm sterile culture dishes.

After 2 days, the cardiomyocytes were treated with PBS (control), T3 (1–3 µM), T3 + PCr (5 µM), PLGA-T3 NPs (1–3 µM), PLGA-T3/PCr NPs (5 µM), and epinephrine (0.5 µM). The cells were cultured in a hypoxia incubator with 4% oxygen, 5% CO_2_ at 37 °C for 24 h and compared with normoxia conditions.

Mitochondrial function, and sarcomere integrity, were studied using an ATP– bioluminescence assay (ATP bioluminescence assay kit HS II, Roche Life Science, Indianapolis, IN, USA) following the manufacturer’s instructions. Mice neonatal cardiomyocytes were incubated with a cell lysis reagent (Sigma, Fremont, CA, USA), and protease inhibitor cocktail (Sigma), for 5 min at room temperature, in darkness. Luciferase reagent was added to the samples, and the luminescence of the live cells was measured by the GloMax luminometer (Promega Corporation, Madison, WI, USA). After adding troponin T antibody conjugated with fluorescein isothiocyanate (FITC) (BD bioscience, San Jose, CA, USA), cardiac troponin T levels were measured using flow cytometry. Confirmation was achieved by immunostaining with anti-troponin T-PE (BD bioscience, USA), using confocal microscopy (LeicaTCS SP5, Wetzlar, Germany). Photographic and fluorescent images were taken at constant exposure time for evaluation of cardiac troponin T levels. Cells were imaged at an excitation wavelength of 488 nm; emission was detected between 505 nm and 560 nm.

### 2.13. Biodistribution of T3 and PLGA-T3 NPs-Cy7

Animal studies were conducted at the animal facility of the Veterans Affairs Medical Center, Albany, NY, USA, and approved by the IACUC committee of the Veterans Affairs Medical Center. Female BALB/c mice aged 5–6 weeks and weighing 18–20 g, were purchased from Taconic Biosciences Inc. (Hudson, NY, USA). Mice were maintained under specific pathogen-free conditions and housed under a controlled temperature (20–24 °C), humidity (60–70%), and 12 h light/dark cycle with access to water and food ad libitum. Mice were allowed to acclimatize for 5 days before the study.

PLGA-NHS and T3 mixture was stirred for at least 24 h. The solution was dialyzed for a minimum of 12 h (3500 molecular weight cut-off), and then 50 μL of Cy7-amine (1 mg/mL in DMSO) was added. The mixture was stirred for an additional 24 h to generate PLGA-T3-Cy7 NPs [24]. The mice were injected (300 µL/mouse) with PLGA-T3-Cy7/PCr NPs and free T3 labeled with Cy7 into the tail vein. Biodistribution of T3 was evaluated immediately at (0), 1, 2, 4 and 24 h using an in vivo imaging system (IVIS^®^, Perkin Elmer, Boston, MA, USA). PBS was injected as the control, and all mice were imaged before injection, to assess the background signals.

### 2.14. Statistical Analysis

An overall comparison of the means for all groups was performed using a one-way ANOVA. Tukey confidence intervals were used to test for differences in means for each experimental group versus the control group. Results are presented as means ± S.D. A value of *p* < 0.05 indicated a statistically significant difference.

## 3. Results

### 3.1. Design, Synthesis and Characterization

Thyroid hormone’s effects on the cardiovascular system make it an attractive therapy for impaired hemodynamics and low levels of T3 [25]. Regulating thyroid hormone levels may be used as a therapeutic option for cardiovascular diseases. Cardioprotection by T3 may be maximized by administering PCr via T3 functionalized NPs, which could also lead to their consistent delivery. Treatment via T3 attached NPs may also change the bio-distribution, and pharmacokinetic profile of T3 and PCr. These NPs may limit T3′s activity to the cell surface and prolong cell membrane-mediated effects. The encapsulation of PCr by PLGA-T3 NPs may enhance the therapeutic effect of T3 by providing additional energy.

**Figure 2:**
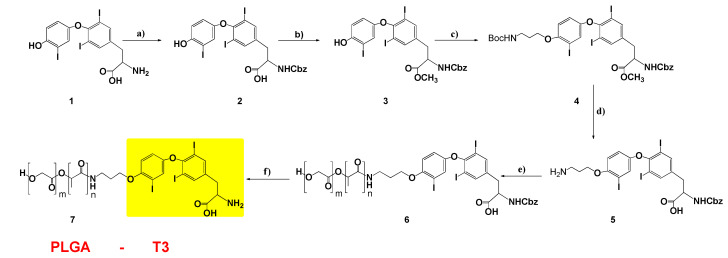
Synthesis of PLGA-T3. (**a**) Benzyl chloroformate, THF;water, rt, 4 h; (**b**) SOCl_2_, 0 °C–70 °C, 2 h; (**c**) tert-Butyl (3-bromopropyl) carbamate, ACN, K_2_CO_3_, reflux, 24 h; (**d**) HCl (4N in dioxane), rt, 24 h; (**e**) PLGA5k-NHS, DMSO, rt, 24 h; and (**f**) HBr (in 33% AcOH), DCM, rt, 24 h.

Based on the above, we designed, and synthesized, new NPs containing T3 and PCr for cardioprotection. Biocompatible, and biodegradable, copolymers of PLGA were used. The first design was to protect T3 on its amine side, and to have suitable linkers for conjugation to the PLGA polymer. Benzyl chloroformate was used to protect the amine group because the benzyloxy carbonyl group attached nitrogen can be cleaved in hydrogen bromide/glacial acetic acid solution, without affecting the polymer PLGA chain [26]. 3-bromo propyl amine was attached to the phenolic oxygen of T3 for polymer linkage, and modified T3 was conjugated to the N-hydroxy succinimide (NHS) activated PLGA polymer (Figure 2).

As shown in Figure 2, the amine and carboxylic acid groups of T3 were protected with benzyl chloroformate and methyl ester, respectively. Compounds **2** and **3** were characterized with ^1^H-NMR and ^13^C-NMR (Figures S1–S4). Compound **3** was reacted with commercially available 3-bromopropyl carbamate in the presence of potassium carbonate and ACN under reflux conditions to make compound **4** with an 87% yield. The ^1^H-NMR spectrum of compound **4** (Figure S5) exhibited triplet peaks of CH_2_ protons at 2.0, 3.4 and 4.0 ppm, which were assigned to the propyl group. The ^13^C-NMR spectrum is also given in Figure S6. Then, BOC-protected propyl amine was deprotected in a 4 N HCl (in dioxane) solution, and the structure of compound **5** was confirmed with both ^1^H and ^13^C-NMR spectra (Figures S7 and S8). 

Compound **5** was conjugated to the PLGA polymer by its free amine group in DMSO. The resulting PLGA-T3-Cbz compound **6** was dialyzed using a membrane (molecular weight cutoff, 10,000 g/mol) for 2 days. The dialyzed compound **6** was lyophilized and dried under vacuum. The peaks of T3 aromatic protons (6.5–8 ppm), and PLGA protons in the ^1^H-NMR spectrum, confirmed the conjugation reaction of compound **7** (Figure S9). Finally, the protecting Cbz group on compound **6** was removed in HBr/CH_3_COOH, and the final product PLGA-T3 (compound **7**) was dialyzed against water using a membrane (MWCO 1kDa) for 2 days. 

### 3.2. Physicochemical Characterization of the Nanoparticles

PLGA-T3 and PLGA-T3/PCr NPs were prepared by spontaneous emulsification solvent diffusion, and modified double emulsion methods, respectively. PLGA is more hydrophobic than T3, and the molecular weight of PLGA is bigger than T3. During the emulsification process of PLGA-T3 in PVA polar medium, it is anticipated that T3 will be directed to the surface of PLGA. The results of particle size distribution show that the average particle sizes were 254 nm, 175 and 178 nm for the void, PLGA-T3 NPs and PLGA-T3/PCr NPs, respectively. The quality of the particle size distribution was measured by the polydisperse index (pdi) these was 0.116, 0.190 and 0.194 for void NPs, PLGA-T3 NPs and PLGA-T3/PCr NPs, respectively. In Table 1, the very low pdi value reflects the high quality of the nano formulation (Figure S10).

**Figure 3:**
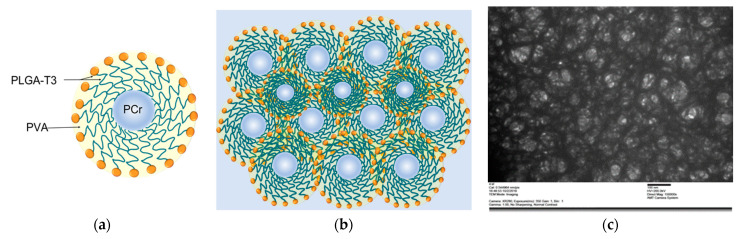
(**a**) Schematic draw of PLGA-T3/PCr nanoparticle. (**b**) Schematic representation of aggregate PLGA-T3/PCr nanoparticles. (**c**) TEM image of PLGA-T3/PCr NPs. TEM image with scale bar 100 nm. revealed the spherical morphology of the PLGA-T3/PCr NPs having an average size of 100–200 nm.

A transmission electron microscopy (TEM) image revealed the spherical morphology of the PLGA-T3/PCr NPs having an average size of 100–200 nm (Figure 3).

### 3.3. Loading Percentage and Entrapment Efficiency

The PCr loading percentage of PLGA-T3/PCr NPs was determined by analyzing the amount of PCr encapsulated in the NPs, compared with the total weight of the PLGA-T3/PCr NPs. After lyophilization, 1 mg of PLGA-T3/PCr NPs powder was dispersed in 1 mL of DMSO for 10 min. Results showed that the PCr loading was 25%, while the entrapment efficiency was 93%. This reflects the success of the preparation method in preventing loss of the active drug.

### 3.4. Release Kinetics

**Figure 4:**
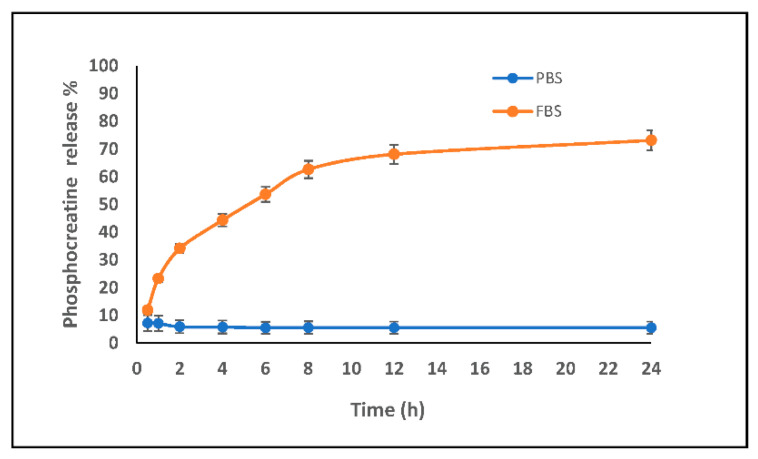
In vitro release kinetics of PCr from PLGA-T3 NPs/PCr in FBS and PBS using HPLC. The release of PCr in PBS was very low, less than 10% for 24 h, compared to FBS, more than 65%, indicating good encapsulation of PCr inside the PLGS-T3 shell.

The release kinetics of the PCr were performed in FBS (fetal bovine serum) and PBS (phosphate buffer saline). A graphical representation comparing the release profiles of PLGA-T3/PCr NPs is shown in Figure 4. Release of the PCr was very low in PBS; at 24 h, only ~7% of the PCr was released. On the other hand, the maximum release in FBS was ~70% after 12 h.

The low release of PCr in PBS might be attributed to the encapsulation of PCr inside the hydrophobic capsule of PLGA hindering the PCr release, while the release was more than 60% in FBS medium (less hydrophilicity than PBS). This confirms the encapsulation of PCr (hydrophilic) inside the PLGA-T3 hydrophobic capsule.

### 3.5. Cardioprotective Effects of T3 and PLGA-T3/PCr NPs under Hypoxia

Under normal conditions (normoxia) neonatal mice cardiomyocytes showed contraction rates and rhythms that were similar for epinephrine, PLGA-T3 NPs and PLGA-T3 NPs/PCr (160–186 contraction/min, regular rhythm, and multiple spots).

Under hypoxic conditions, epinephrine showed no improvement in cardiomyocyte function (28–39 contractions/min, irregular rhythm, and random spots). On the other hand, PLGA-T3 NPs and PLGA-T3 NPs/PCr showed significant improvement (134–145, contractions/min, regular rhythm, and multiple spots) (*p* < 0.001 versus untreated).

**Figure 5:**
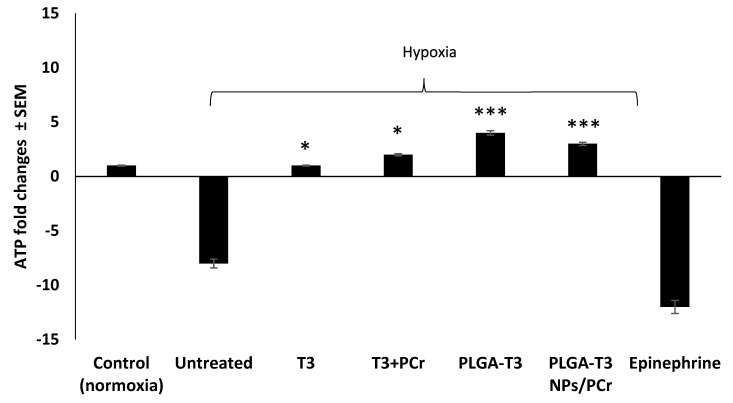
Cardioprotective effect of T3, PLGA-T3 NPs with or without PCr in normoxia vs. hypoxic conditions. ATP levels in neonatal cardiomyocytes (bioluminescence assay), * *p* < 0.01 versus hypoxia. * *p* < 0.01, *** *p* < 0.001 versus hypoxia (control untreated), with full reversal and improvement versus control under normoxic condition.).

Since ATP synthesis depends on the mitochondrial membrane potential, we subsequently tested to see whether PLGA-T3 NPs and PLGA-T3 NPs/PCr could protect this membrane potential. PLGA-T3 NPs and PLGA-T3 NPs/PCr significantly protected the degradation of ATP (~5 folds increase in ATP levels) (*p* < 0.01 versus T3 and T3+PCr) (Figure 5). Epinephrine was associated with a marked degeneration of ATP levels. These data indicate that PLGA-T3 NPs and PLGA-T3 NPs/PCr may improve glycolysis and suppress the abnormal accumulation of mitochondrial reactive oxygen species.

**Figure 6:**
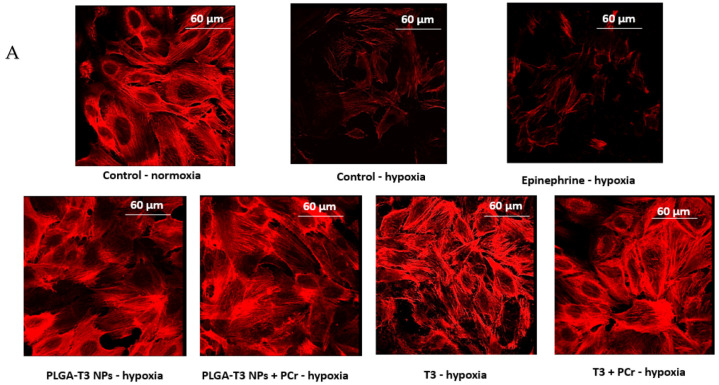

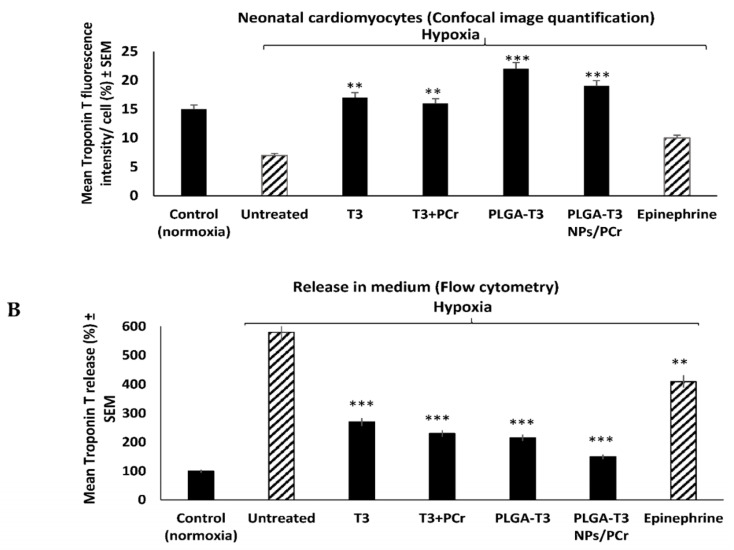
Expression of troponin T in neonatal cardiomyocytes under hypoxic condition. (**A**) Immunostaining by anti-troponin T-PE (confocal microscopy). (**B**) Quantitation of troponin T in neonatal cardiomyocyte and in medium. ** *p* < 0.05, *** *p* < 0.001 versus control untreated, with full reversal and improvement versus control under normoxic condition.These results suggest that of PLGA-T3 NPs and PLGA-T3 NPs/PCr inhibit the damaging effect of hypoxia on cardiomyocytes.

Similarly, under hypoxic conditions with PLGA-T3 NPs and PCr encapsulated PLGA-T3 NPs, troponin T levels released into the medium were decreased 3-fold (** *p* < 0.05, and *** *p* < 0.001, respectively; Figure 6B). The results were confirmed with the confocal microscopy (Figure 6A).

### 3.6. Evaluation of Free T3 and PLGA-T3 NPs-Cy7 Biodistribution in Mice

**Figure 7:**
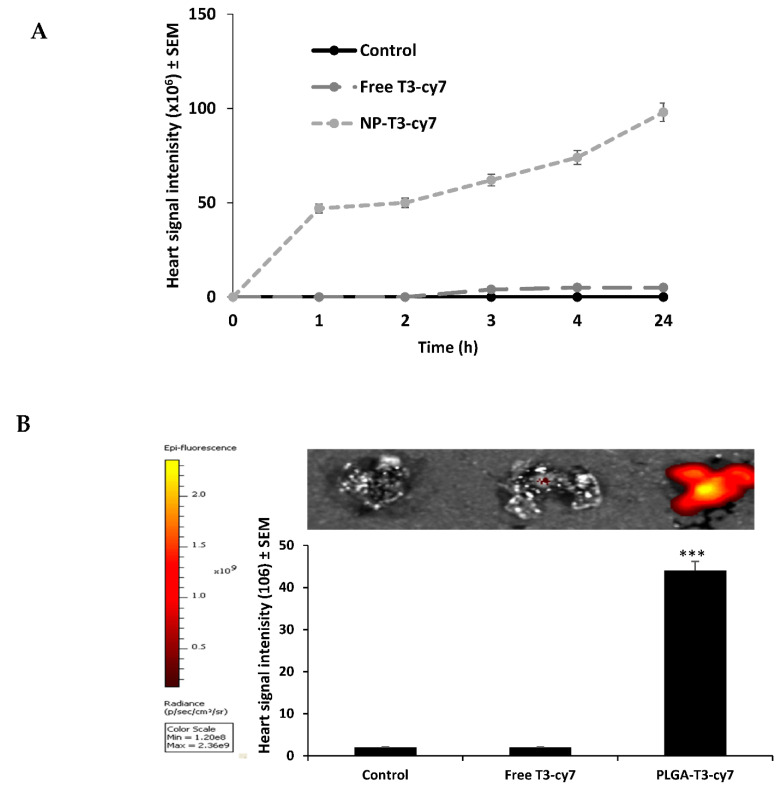
Free T3-Cy7 and PLGA-T3-Cy7 biodistribution in heart in mice (BALB/c) after injection of 300 uL subcutaneously. Cy7 signals detected with IVIS at excitation/emission maximum 750/776 nm wavelength. IVIS images taken to detect Cy7 signals during the following time: (**A**) Before injection, immediately, 1, 2, 3, 4 and 24 h. (**B**) Sacrificed at 24 h. Heart Cy7 signals imaged ex vivo, *** *p* < 0.001 versus control and T3-cy7.

The Cy7 signal intensity of PLGA-T3 NPs-Cy7 were detected as strong signals within 5 min of administration, primarily in mice hearts (Figure 7). After 24 h, the Cy7 signal intensity of PLGA-T3 NPs-Cy7 was about 40–50-fold, compared to the control and free T3 indicating the successful delivery to the heart tissue.

## 4. Discussion

Several studies have shown that thyroid hormones play a crucial role in cardioprotection after HF because of their effects on molecular pathways and cardiac function and structure. PLGA-T3, a nanoparticle which incorporates T3, may be the first of a new class of therapeutic agents. In this study, we aimed to develop T3 conjugated PLGA-T3 NP to facilitate the active targeting of thyrointegrin αvβ3 receptors on the cell surface/cytoplasm and restrict its nuclear translocation. It utilizes the biological properties inherent in the relationship between T3 and its αvβ3 membrane receptor [27], and preferentially attaches to the receptor in hypoxic cells. The hypoxic and acidic medium of the ischemic heart may be a reason for the enhanced NP degradation, and drug release [28,29]. Our in vitro study indicates that PLGA-T3 stimulate angiogenesis as T3 itself, which shows that T3 is still active after conjugation with PLGA. Additionally, we confirmed PCr encapsulation after nano preparation with HPLC, which refers to the existence of active PCr (data not shown).

The major activity of PLGA-T3/PCr NPs appears to be the delivery of energy to an energy depleted cell. In cardiac arrest (CA), oxidative phosphorylation stops and, as a result, both ATP and PCr, critical contributors to cellular energy utilization, are depleted within a few minutes [30,31,32]. PLGA-T3/PCr NPs may provide energy to the cell, until normal oxidative phosphorylation can be restored. 

PLGA-T3/PCr NPs can improve the energy status in two ways. The first is initiated by PLGA-T3/PCr NP itself. It is configured to present T3 to the membrane receptor αvβ3 in such a manner that only the T3’s non-genomic properties are activated [33]. This activation provides the energy to activate a series of plasma membrane ion pumps. T3 is an important regulator of several cell surface ion pumps crucial to cardiac resuscitation. These include Na^+^/K^+^-ATPase, the Na^+^/H^+^ antiporter and Ca^+2^-ATPase. The activation of these pumps restores cell membrane electrical potentials, thus initiating cell contraction [34]. This action also introduces energy into the cytoplasm to help restore mitochondrial activity [35]. The second is the incorporation of PCr within the architecture of PLGA-T3/PCr NP. When the PCr released upon contact with the αvβ3 receptor [36,37], it is immediately carried into the cell cytoplasm where creatine kinase, located mainly in the cristae of the plasma membrane of the mitochondria [38], play an important role in the regulation of the cellular energy homeostasis. The phosphate delivers energy to the cell by phosphorylation of ADP to make ATP, as well as other phosphate dependent reactions involved in the delivery of energy within the cell [39]. Delivering PCr to the cell fulfills many of its acute energy needs, thereby facilitating the cell’s survival. 

Forini et al., considered the cardio protective effects of T3 in ischemic condition. They showed that T3 treatment can prevent further mitochondrial disturbances and damage, in ischemic cases, and thoroughly described the T3 cardio protective effects, particularly on oxidative stress conditions [8,9]. Similarly, Lin et al. demonstrated that T3, but not T4, increases Src/PI3K phosphorylation through integrin αvβ3 in human glioma cells [40]. PLGA-T3/PCr NPs may elicit their actions by a non-classical mechanism, without direct gene transcription regulation by nuclear Thyroid Receptors (TRs). These non-genomic actions of PLGA-T3/PCr NPs may be mediated indirectly by modulating gene function. Furthermore, consistent with our results, regarding the rapid distribution of PLGA-T3-Cy into the brain, heart and lung tissue, Davis et al. discussed the neuroprotective effects of thyroid hormone including the upregulation of the seladin-1 gene, which is associated with antiapoptotic activity [41].

In addition, the T4 serum transport protein, transthyretin, was found to be neuroprotective in the setting of brain ischemia indicating that it rapidly penetrates the blood brain barrier. TTR (Transthyretin) gene expression was also reported to be upregulated in ischemic brain tissue, in TTR null mice [41].

Finally, our data has shown that PLGA-T3 NPs showed distinct improvement in all points measures regarding cardioprotective effect; mitochondrial function (ATP, LDH) and myocardial integrity (troponin level) against the current standard of care (epinephrine). Therefore, PLGA-T3 NPs may have advantages over epinephrine for ischemic heart disease, not only by its cardioprotective effects, but also by its neuroprotective effects.

## Figures and Tables

**Table 1:** Average particle size and pdi results of the nanoformulations.

Nanoformulation	Z-Average Size (d.nm)	Polydisperse Index
Void NPs	254	0.116
PLGA-T3 NPs	175	0.19
PLGA-T3/PCr NPs	176	0.194

## Data Availability

Additional figures showing ^1^H-NMR and ^13^C-NMR spectra of the synthesized compounds.

## Supplementary Materials

The following are available online at https://www.mdpi.com/2227-9059/9/11/1713/s1, Figure S1. 1H-NMR spectrum of compound **2**, Figure S2. 13C-NMR spectrum of compound **2**, Figure S3. 1H -NMR spectrum of compound **3**, Figure S4. 13C-NMR spectrum of compound **3**, Figure S5. 1H -NMR spectrum of compound **4**, Figure S6. 13C -NMR spectrum of compound **4**, Figure S7. 1H -NMR spectrum of compound **5**, Figure S8. 13C -NMR spectrum of compound **5**, Figure S9. 1H -NMR spectrum of compound **7**, Figure S10. Characterization of prepared nano capsules (a) particle size distribution of void nanoparticles showing average size of 150 nm; (b) particle size of PLGA-T3 NPs; (c) particle size of PLGA-T3/PCr NPs, Figure S11. Calibration curve of T3.
