# Correction: Karakus et al. Development of Triiodothyronine Polymeric Nanoparticles for Targeted Delivery in the Cardioprotection against Ischemic Insult. *Biomedicines* 2021, *9*, 1713

**DOI:** 10.3390/biomedicines10061395

**Published:** 2022-06-13

**Authors:** Ozlem Ozen Karakus, Noureldien H. E. Darwish, Thangirala Sudha, Taher A. Salaheldin, Kazutoshi Fujioka, Peter C. Taylor Dickinson, Brian Weil, Shaker A. Mousa

**Affiliations:** 1 The Pharmaceutical Research Institute, Albany College of Pharmacy and Health Sciences, Rensselaer, NY 12144, USA; ozlem.karakus@acphs.edu (O.O.K.); noureldien.darwish@acphs.edu (N.H.E.D.); sudha.thangirala@acphs.edu (T.S.); taher.salaheldin@acphs.edu (T.A.S.); kazutoshi.fujioka@acphs.edu (K.F.); 2 Clinical Pathology (Hematology Section), Faculty of Medicine, Mansoura University, Mansoura 35516, Egypt; 3 Pro-Al Medico Technologies Inc., Suffern, NY 10901, USA; dickinsonpct@gmail.com; 4 Departments of Physiology & Biophysics, School of Medicine & Biomedical Sciences, University at Buffalo, Buffalo, NY 14203, USA; bweil@buffalo.edu; 5 VA WNY Healthcare System, Buffalo, NY 14215, USA

In the published manuscript [1] in the Results section, in the subsection titled 3.5. Cardioprotective Effects of T3 and PLGA-T3/PCr NPs under Hypoxia, in Figure 6A there was an unintentional mistake of an overlap in Figures under different treatment conditions while taking photographs/labeling at a lower magnification. Experiment was repeated and photographs were taken under higher magnification for improved image quality. The scientific conclusion was unaffected and there is no change in the legend of the figure. The published figure is replaced with the new Figure 6A.

The authors apologize for any inconvenience. This correction was approved by the Academic Editor. The original publication has also been updated.

## Figures and Tables

**Figure 6:**
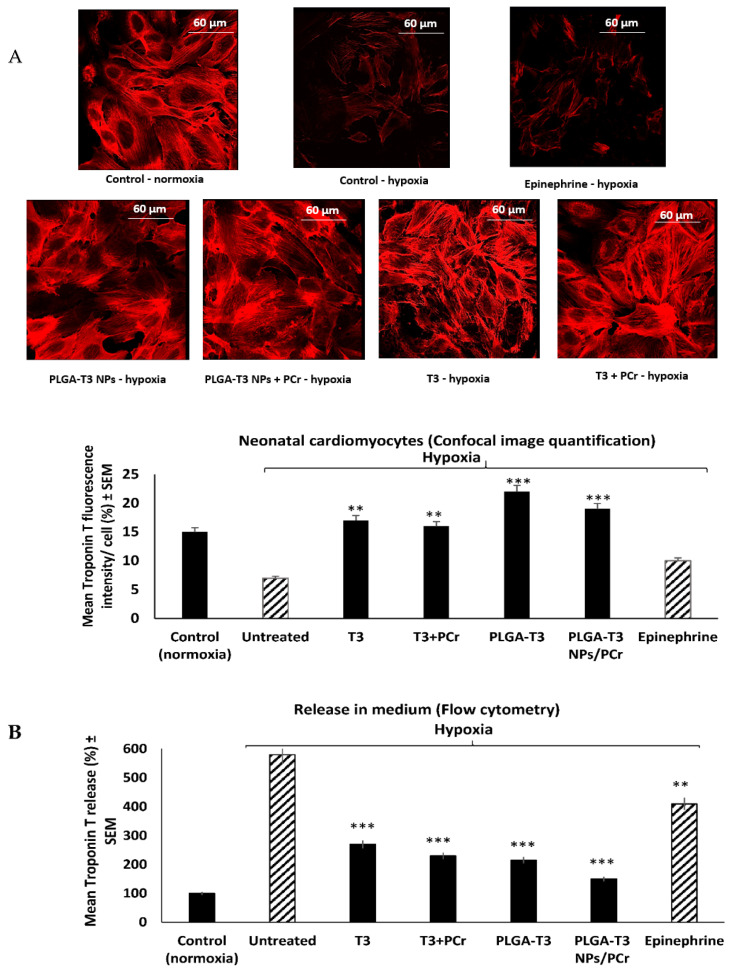
Expression of troponin T in neonatal cardiomyocytes under hypoxic condition. (**A**) Immunostaining by anti-troponin T-PE (confocal microscopy). (**B**) Quantitation of troponin T in neonatal cardiomyocyte and in medium. ** *p* < 0.05, *** *p* < 0.001 versus untreated control, with full reversal and improvement versus control under normoxic condition. These results suggest that PLGA-T3 NPs and PLGA-T3 NPs/PCr inhibit the damaging effect of hypoxia on cardiomyocytes.
