# Supplementary material for: Development of Triiodothyronine Polymeric Nanoparticles for Targeted Delivery in the Cardioprotection against Ischemic Insult
Source: Biomedicines. 2021 Nov 18;9(11):1713. doi: 10.3390/biomedicines9111713 (PMC8615924; doi:10.3390/biomedicines9111713)
Supplement: Supplementary file 1 [file biomedicines-09-01713-s001.zip › biomedicines-1434866-supplementary/biomedicines-1434866-SM.pdf]

Development of Triiodothyronine Polymeric Nanoparticles for Targeted Delivery in the Cardioprotection against Ischemic Insult

Ozlem O. Karakus [1], Noureldien H. E. Darwish [1,2], Thangirala Sudha [1], Taher A. Salaheldin [1], Kazutoshi Fujioka [1], P. C. Taylor Dickinson [3], Brian Weil [4,5] and Shaker A. Mousa [1,*]

[1] The Pharmaceutical Research Institute, Albany College of Pharmacy and Health Sciences, Rensselaer, NY 12144, USA; Ozlem.Karakus@acphs.edu (O.O.K.); noureldien.darwish@acphs.edu (N.H.E.D.); sudha.thangirala@acphs.edu (T.S.); taher.salaheldin@acphs.edu (T.A.S.); Kazutoshi.Fujioka@acphs.edu (K.F.)
[2] Clinical Pathology (Hematology Section), Faculty of Medicine, Mansoura University, Mansoura 35516, Egypt
[3] Pro-Al Medico Technologies Inc., Suffern, NY 10901, USA; dickinsonpct@gmail.com
[4] Departments of Physiology & Biophysics, School of Medicine & Biomedical Sciences, University at Buffalo, Buffalo, NY 14203, USA; bweil@buffalo.edu
[5] VA WNY Healthcare System, Buffalo, NY 14215, USA

***Correspondence**:

Shaker A. Mousa
The Pharmaceutical Research Institute
Albany College of Pharmacy and Health Sciences
1 Discovery Drive, Rensselaer, NY 12144
Phone: (518) 694-7397
Fax: (518) 694-7567
Email: shaker.mousa@acphs.edu

Table of Contents

Figure S1. ^{1}H-NMR spectrum of compound 2

Figure S2. ^{13}C-NMR spectrum of compound 2

Figure S3. ^{1}H -NMR spectrum of compound 3

Figure S4. ^{13}C-NMR spectrum of compound 3

Figure S5. ^{1}H -NMR spectrum of compound 4

Figure S6. ^{13}C -NMR spectrum of compound 4

Figure S7. ^{1}H -NMR spectrum of compound 5

Figure S8. ^{13}C -NMR spectrum of compound 5

Figure S9. ^{1}H -NMR spectrum of compound 7

Figure S10. Characterization of prepared nano capsules (a) particle size distribution of void nanoparticles showing average size of 150 nm; (b) particle size of PLGA-T3 NPs; (c) particle size of PLGA-T3/PCr NPs

Figure S11. Calibration curve of T3

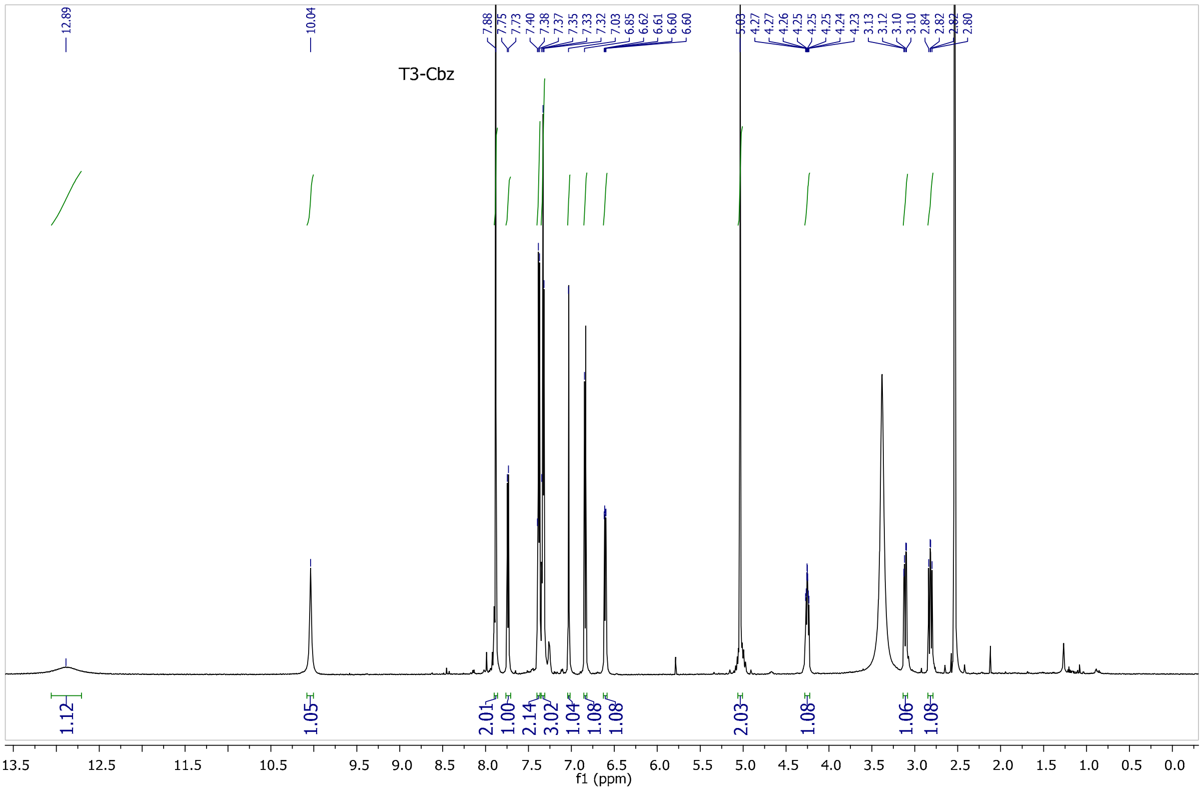

Figure S1: ^{1}H-NMR Spectrum of compound 2

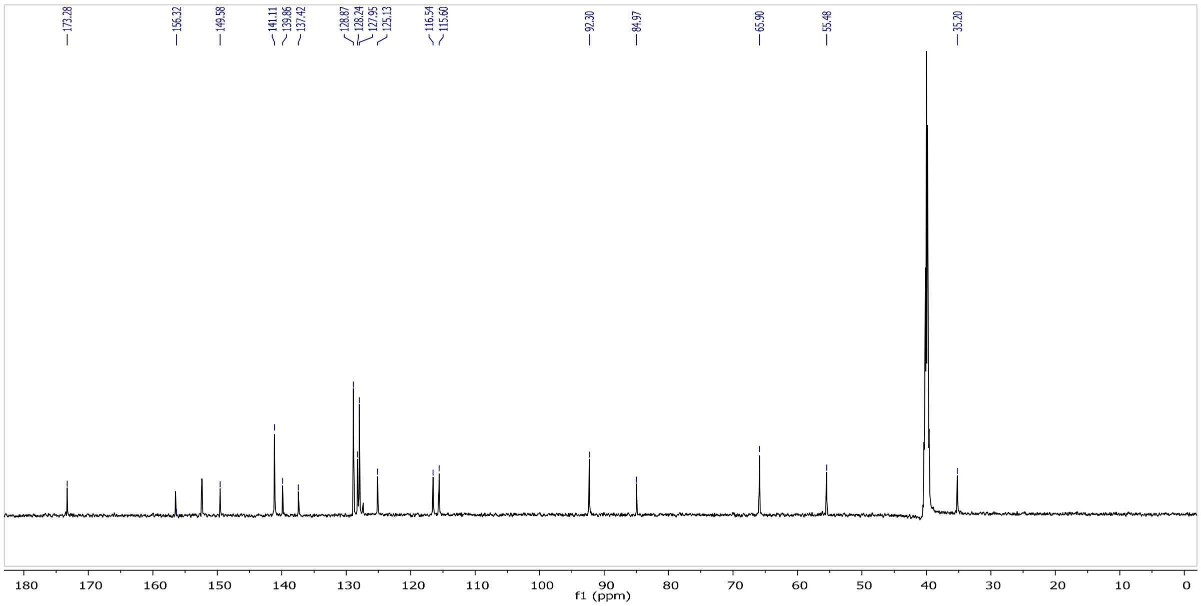

Figure S2: ^{13}C-NMR Spectrum of compound 2

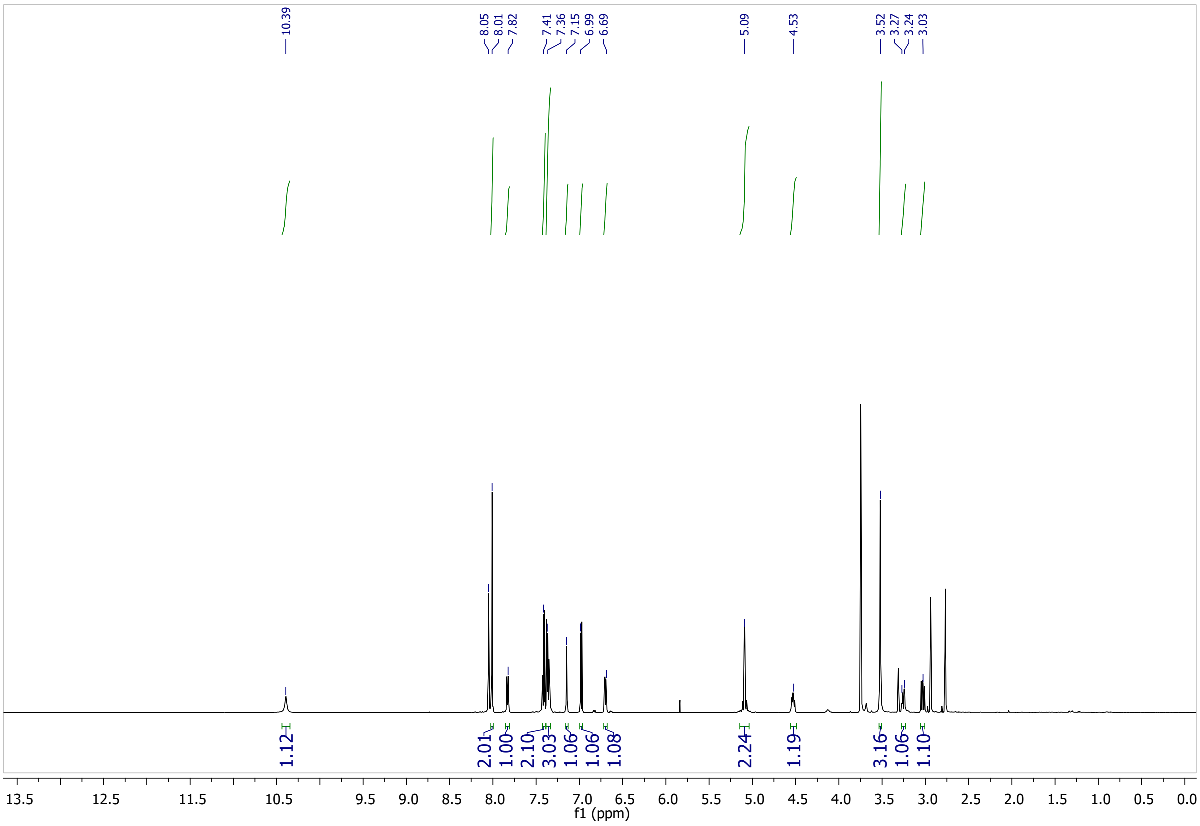

Figure S3: ^{1}H-NMR Spectrum of compound 3

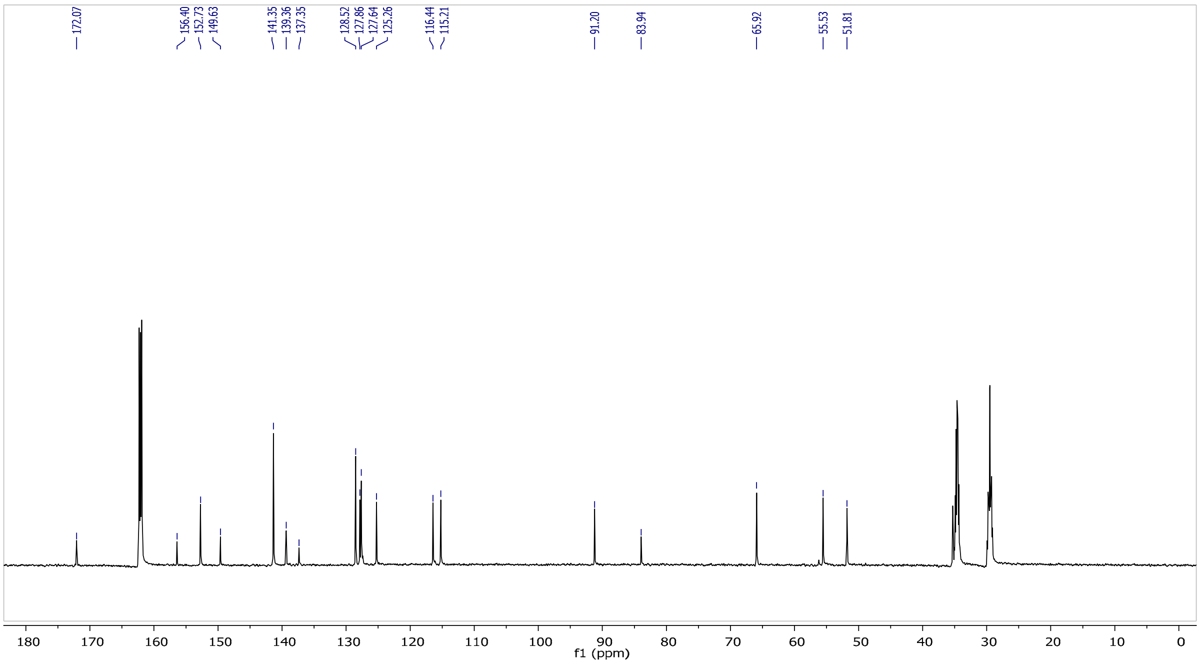

Figure S4: ^{13}C-NMR Spectrum of compound 3

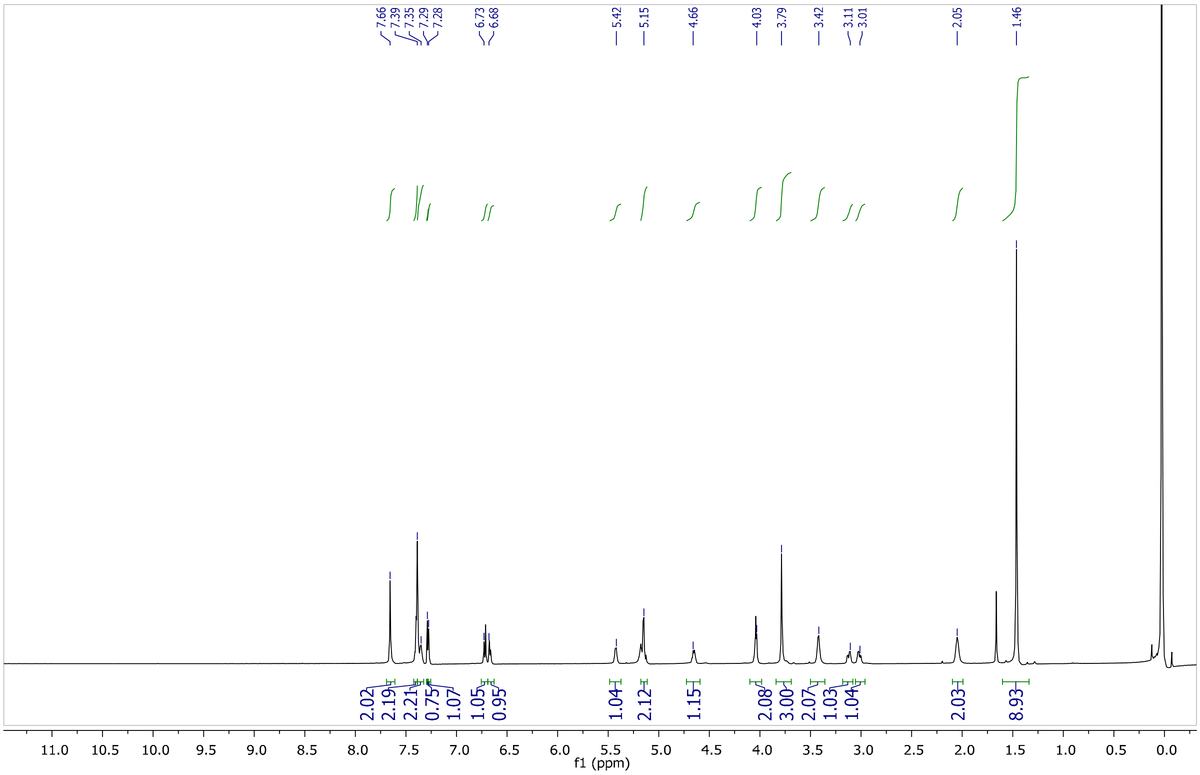

Figure S5: ^{1}H-NMR Spectrum of compound 4

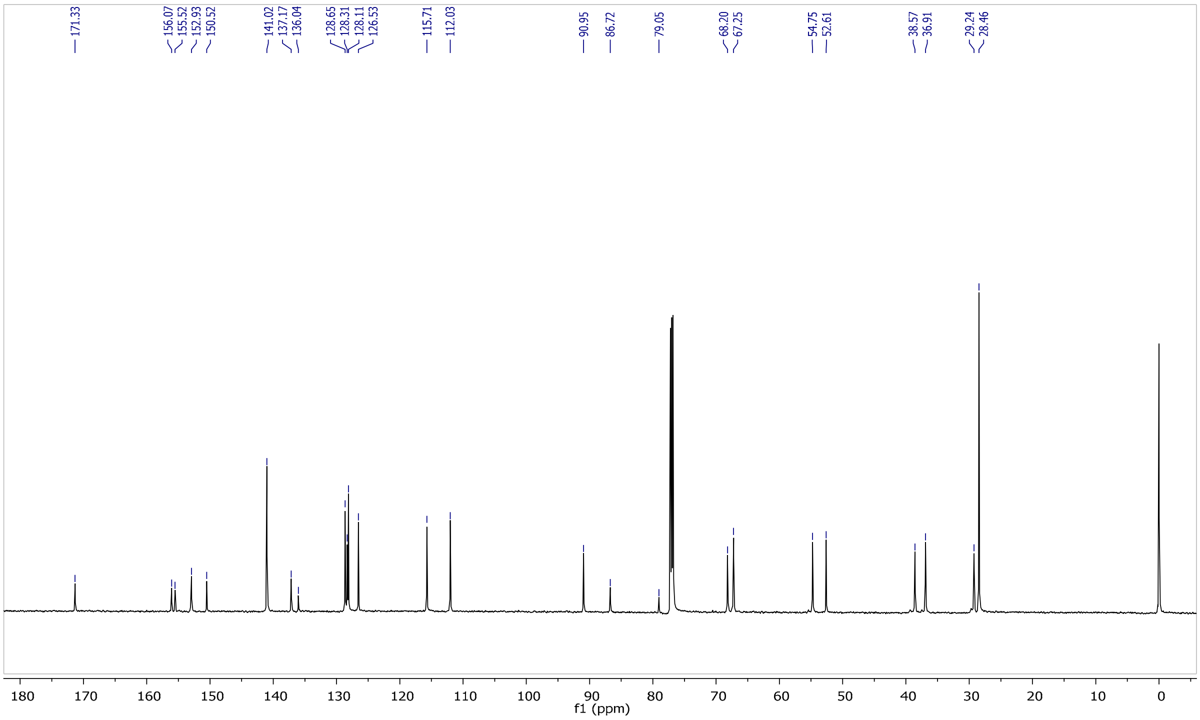

Figure S6: ^{13}C-NMR Spectrum of compound 4

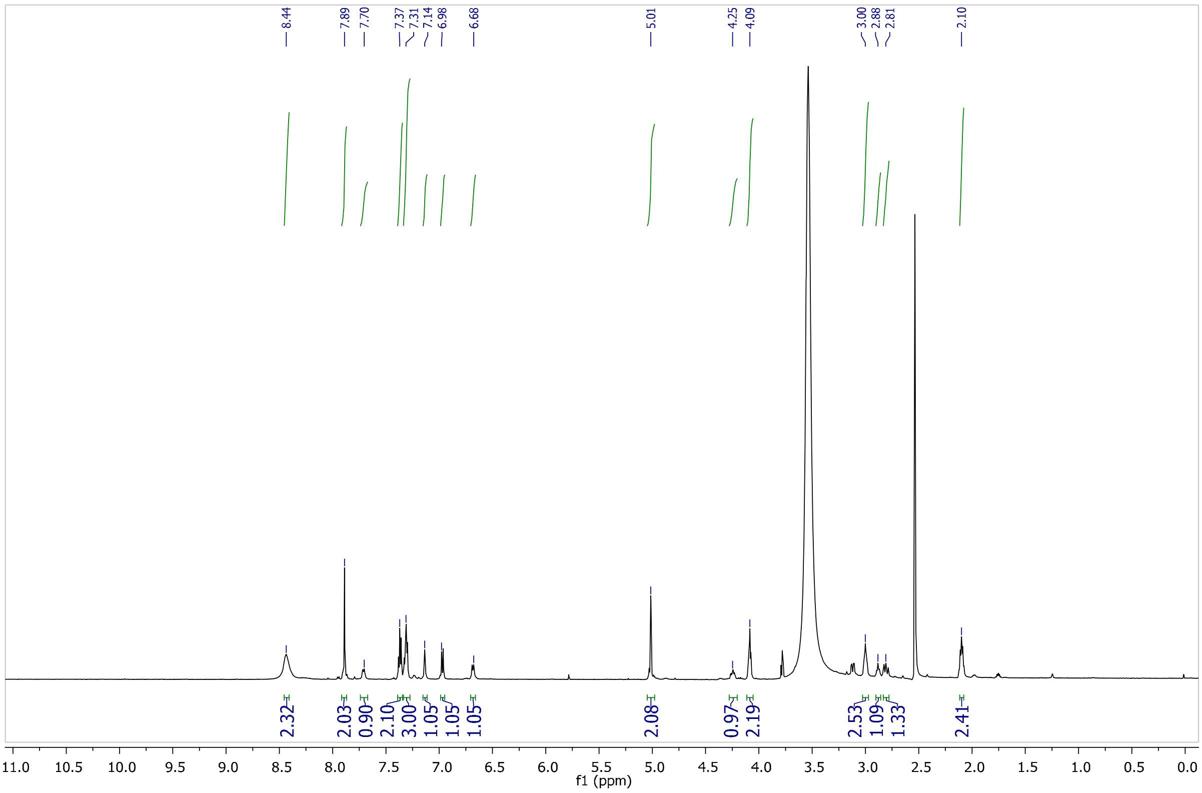

Figure S7: ^{1}H-NMR Spectrum of compound 5

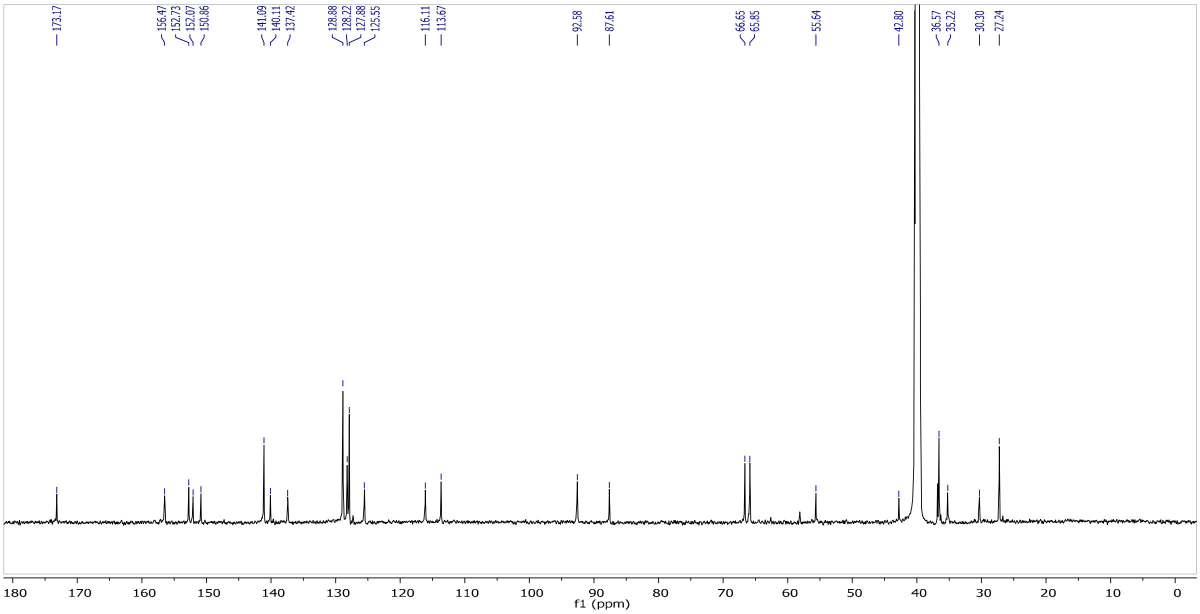

Figure S8: ^{13}C-NMR Spectrum of compound 5

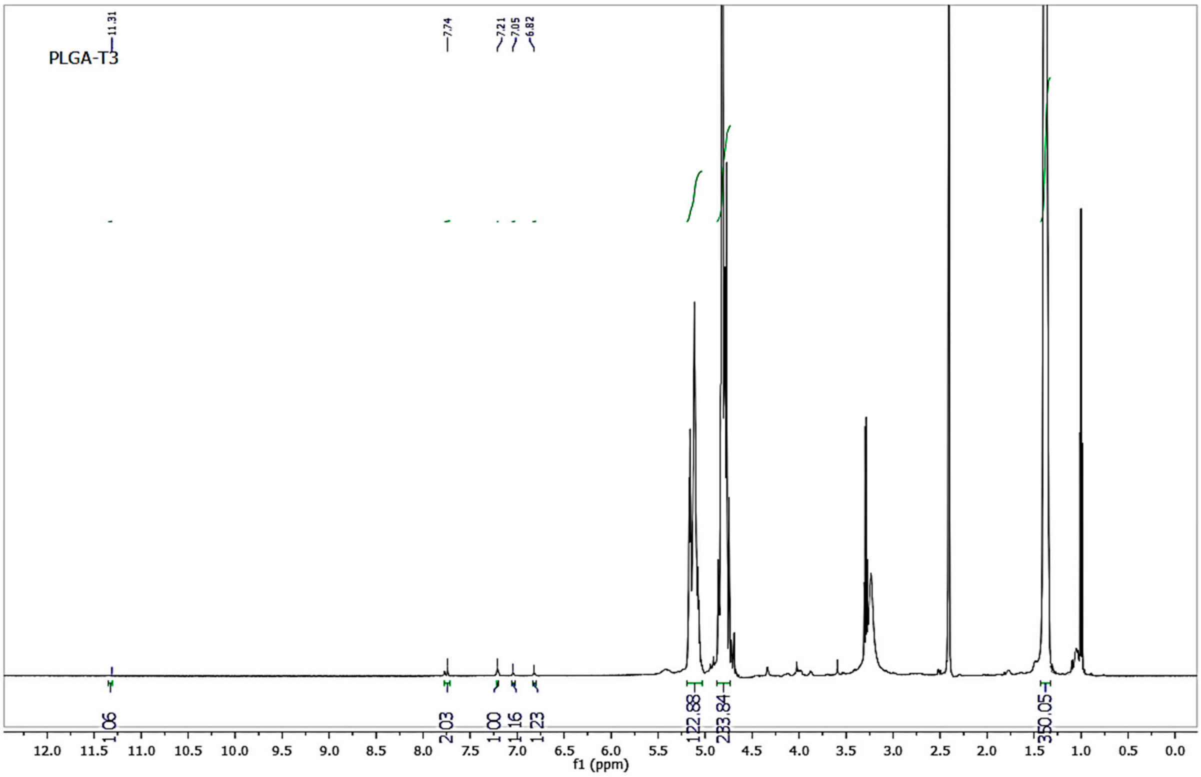

Figure S9: 1H-NMR Spectrum of compound 7

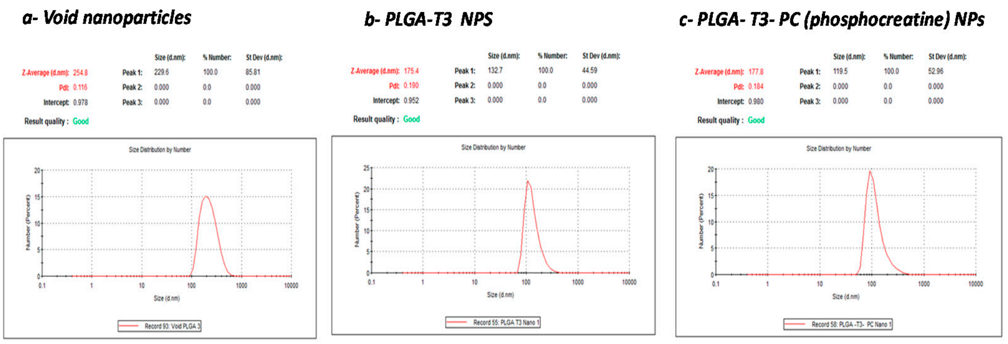

Figure S10. Characterization of prepared nano capsules (a) particle size distribution of void nanoparticles showing average size of 150 nm (250?); (b) particle size of PLGA-T3 NPs, size 175; (c) particle size of PLGA-T3/PCr NPs, size 187.

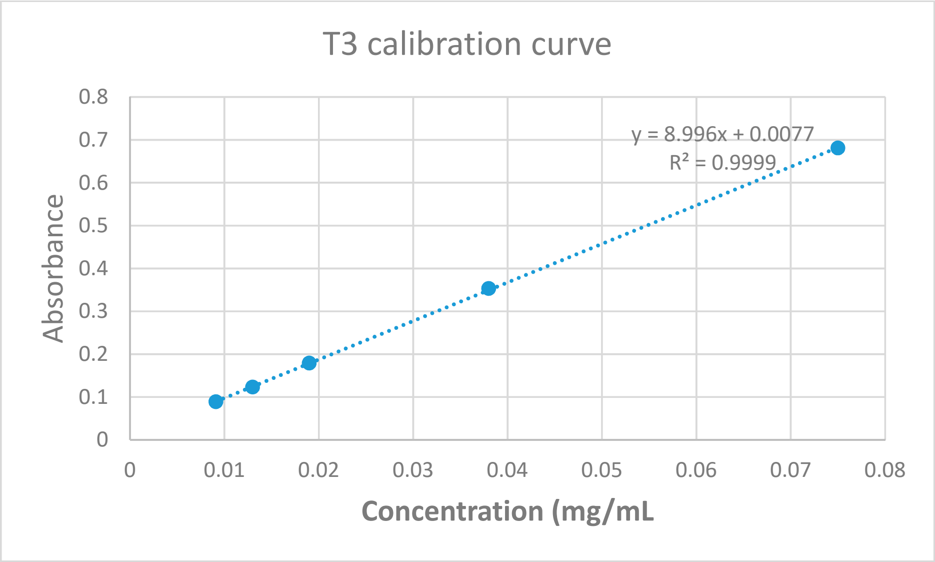

Figure S11. Calibration curve of T3
